# Cerebral Hypoperfusion Intensity Ratio Is Linked to Progressive Early Edema Formation

**DOI:** 10.3390/jcm11092373

**Published:** 2022-04-23

**Authors:** Noel van Horn, Gabriel Broocks, Reza Kabiri, Michel C. Kraemer, Soren Christensen, Michael Mlynash, Lukas Meyer, Maarten G. Lansberg, Gregory W. Albers, Peter Sporns, Adrien Guenego, Jens Fiehler, Max Wintermark, Jeremy J. Heit, Tobias D. Faizy

**Affiliations:** 1 Department of Diagnostic and Interventional Neuroradiology, University Medical Center Hamburg-Eppendorf, 20246 Hamburg, Germany; no.vanhorn@uke.de (N.v.H.); g.broocks@uke.de (G.B.); reza.kabiri@live.de (R.K.); michel_1@hotmail.de (M.C.K.); lu.meyer@uke.de (L.M.); fiehler@uke.de (J.F.); 2 Department of Neurology and Neurological Sciences, Stanford University School of Medicine, Stanford, CA 94305, USA; sorenc@stanford.edu (S.C.); mmlynash@stanford.edu (M.M.); lansberg@stanford.edu (M.G.L.); albers@stanford.edu (G.W.A.); 3 Department of Diagnostic and Interventional Neuroradiology, University Hospital Basel, 4031 Basel, Switzerland; peter.sporns@usb.ch; 4 Department of Interventional Neuroradiology, Erasme University Hospital, 1070 Brussels, Belgium; adrien.guenego@erasme.ulb.ac.be; 5 Department of Radiology, Stanford University School of Medicine, Stanford, CA 94305, USA; max.wintermark@gmail.com (M.W.); jeremyheit@gmail.com (J.J.H.)

**Keywords:** brain edema, collateral circulation, ischemic stroke, perfusion imaging, thrombectomy

## Abstract

The hypoperfusion intensity ratio (HIR) is associated with collateral status and reflects the impaired microperfusion of brain tissue in patients with acute ischemic stroke and large vessel occlusion (AIS-LVO). As a deterioration in cerebral blood flow is associated with brain edema, we aimed to investigate whether HIR is correlated with the early edema progression rate (EPR) determined by the ischemic net water uptake (NWU) in a multicenter retrospective analysis of AIS-LVO patients anticipated for thrombectomy treatment. HIR was automatically calculated as the ratio of time-to-maximum (TMax) > 10 s/(TMax) > 6 s. HIRs < 0.4 were regarded as favorable (HIR+) and ≥0.4 as unfavorable (HIR−). Quantitative ischemic lesion NWU was delineated on baseline NCCT images and EPR was calculated as the ratio of NWU/time from symptom onset to imaging. Multivariable regression analysis was used to assess the association of HIR with EPR. This study included 731 patients. HIR+ patients exhibited a reduced median NWU upon admission CT (4% (IQR: 2.1–7.6) versus 8.2% (6–10.4); *p* < 0.001) and less median EPR (0.016%/h (IQR: 0.007–0.04) versus 0.044%/h (IQR: 0.021–0.089; *p* < 0.001) compared to HIR− patients. Multivariable regression showed that HIR+ (β: 0.53, SE: 0.02; *p* = 0.003) and presentation of the National Institutes of Health Stroke Scale (β: 0.2, SE: 0.0006; *p* = 0.001) were independently associated with EPR. In conclusion, favorable HIR was associated with lower early edema progression and decreased ischemic edema formation on baseline NCCT.

## 1. Introduction

Cerebral hypoperfusion below a critical threshold of 10 to 15 mL blood/100 g brain tissue per minute may result in ischemic tissue damage and subsequent brain edema formation [1,2,3]. Brain edema formation is one of the critical hallmarks for the prognostication of clinical and procedural outcomes of AIS-LVO patients [1,4]. Aggravated brain edema development is inherently linked to persistent critical hypoperfusion, and extensive edema formation was found to be associated with poor clinical outcomes and the occurrence of malignant infarction, despite successful endovascular treatment [1,3,5]. Thus, a proper and reliable assessment of tissue edema may have strong implications for treatment triage in stroke patients.

Computed tomography perfusion imaging offers an estimation of cerebral blood perfusion over time. The time when the residue function reaches its peak (TMax) is a parameter broadly utilized for infarct core volume and penumbra tissue estimation. The hypoperfusion intensity ratio (HIR) is automatically calculated from perfusion imaging source data as the ratio of the time-to-maximum of the tissue residue function: (TMax) > 10 s/(TMax) > 6 s [6]. Thereby, HIR also reflects the fraction of more severely infarcted brain tissue. Favorable HIR profiles are linked to robust tissue collateral status, lower baseline core volumes, higher penumbra volumes, and also to good functional outcomes after thrombectomy treatment [7,8,9,10]. However, whether HIR status is associated with time-dependent early edema progression and quantitative brain edema estimates on admission imaging has not been investigated.

The purpose of this study was to assess whether HIR may serve as a standardized and reliable imaging biomarker for the assessment of early brain edema progression in AIS-LVO patients anticipated for thrombectomy treatment. We hypothesized that favorable HIR profiles are associated with less temporal ischemic lesion growth from symptom onset to imaging, and decreased quantitative ischemic lesion net water uptake (NWU) on baseline non-contrast head computed tomography (NCCT) compared to patients who exhibited unfavorable HIR profiles.

## 2. Materials and Methods

### 2.1. Study Design

We conducted a multicenter cohort study of consecutive AIS-LVO patients undergoing thrombectomy triage at two comprehensive stroke centers (redacted). Patients were retrospectively screened for inclusion from a prospectively maintained stroke database between January 2013 and December 2020. The study protocol was approved by the institutional review boards of both study centers, and complied with the Health Insurance Portability and Accountability Act (HIPAA) and followed the guidelines of the Declaration of Helsinki. Informed patient consent was waived by the review boards for this retrospective study.

### 2.2. Patient Inclusion, Population, and Clinical Data

Clinical, imaging, and demographic data were obtained from the electronic medical records of each patient.

Inclusion criteria were as follows: (1) Patients with suspected AIS-LVO who underwent thrombectomy triage with the intention-to-treat by endovascular measures within 16 h after stroke onset; (2) available baseline multimodal head CT including NCCT, CT angiography, and CT perfusion imaging; (3) known time of symptom onset to imaging (in minutes); and (4) presence of anterior circulation large vessel occlusion of the internal carotid artery or first (M1) or second (M2) segment of the middle cerebral artery. Exclusion criteria were as follows: (1) poor admission CT image quality due to excessive patient motion or failed contrast bolus, (2) detection of parenchymal hemorrhage on baseline NCCT imaging that impedes delineation of ischemic lesion NWU, and (3) unknown time of symptom onset (e.g., in the scenario of a wake-up stroke).

### 2.3. Imaging Analysis

All CT perfusion studies were automatically analyzed with RAPID (iSchemaView, Menlo Park, CA, USA). The image analyzation software Horos (Horos Project©, v 3.3.6) was used to assess all of the NCCT and CT angiography images, including NWU determination.

The ischemic core was defined as the volume of tissue with ≥70% reduction in cerebral blood flow relative to the contralateral hemisphere on CT perfusion.

HIR was defined as the volume of ischemic brain tissue with a time-to-maximum of the residue function (TMax) delay of >10 s divided by the volume of brain tissue with a TMax delay of >6 s [11]. A favorable HIR was regarded as a ratio of ≤0.4, and unfavorable HIR was defined as >0.4 based on previously published thresholds for this multicenter stroke cohort, as published in [12].

The Alberta Stroke Program Early CT Score (ASPECTS) [13] was determined on pre-treatment NCCT images by two neuroradiologists (T.D.F. and J.J.H.) on a subset of studies to determine the inter-reader agreement, and then the remaining studies were assigned by a single neuroradiologist (T.D.F.) with 10 years of experience.

Pial arterial collateral status was determined on CT angiography in a manner identical to ASPECTS assessment using the modified Tan scale [14], and robust collaterals were defined as filling of ≥50% of the middle cerebral artery (MCA) territory, whereas poor collaterals filled <50% of the MCA territory.

Ischemic lesion NWU was determined by the approach reported by Broocks et al. and Minnerup et al. [1,15]. In brief, ischemic lesion hypodensity was evaluated on admission NCCT images and CT perfusion images were used to verify the area of tissue infarction. Next, ischemic lesion hypodensities were outlined defining a region of interest, which was mirrored to the contralateral unaffected hemisphere. Manual adjustments to the region of interest were made to exclude the sulci and cerebrospinal fluid, if applicable. A histogram was sampled from the region of interest for Houndsfield units between 20 and 80. NWU was calculated in %, as described by Broocks et al. [1].

The early edema progression rate (EPR) was calculated as the ratio of the time from symptom onset to imaging (per hour) divided by ischemic lesion NWU (in %) determined on admission NCCT [16].

### 2.4. Outcome Measures

The primary study outcome was EPR (in %/h). Functional outcomes were assessed by the modified Rankin scale at 90 days (mRS90), and good outcomes were regarded as mRS90 of 0–2.

### 2.5. Statistical Analysis

All data are presented as mean ± standard deviation or median and interquartile range (IQR). The Kolmogorov–Smirnov test was used to test for normal or non-normal distribution. Absolute and relative frequencies are given for categorical data. Univariate regression analysis was performed to compare the clinical, radiological, and outcome parameters for patients with favorable and unfavorable HIR profiles using the chi-square test for counts and the Mann–Whitney U test for measurements. For the logistic regression models, the proportional-odds assumption had to be met (*p* < 0.05) before further analysis. Tests for collinearity with regards to HIR were applied to the included variables. Multivariable linear logistic regression analysis was performed to estimate the independent impact of favorable HIR on EPR. This model was adjusted for variables showing significance in the univariate analysis, i.e., presentation NIHSS, baseline ASPECTS, favorable TAN collaterals, and proximal vessel occlusion, as well as for additional variables that did show a significant association with early edema development or progression in previous studies, such as age, sex, and baseline glucose, although these variables did not show a significant difference between the HIR groups in the univariate analyses (Table 1). Statistical significance was set at the level of *p* = 0.05. A statistical analysis of all data was performed using SPSS (IBM Corp. Released 2016. IBM SPSS Statistics for Windows, Version 24.0. Armonk, NY, USA) and the StataCorp. (2019. Stata Statistical Software: Release 16. StataCorp LLC: College Station, TX, USA). Any unknown data are indicated in the given section below the tables.

## 3. Results

**Figure 1:**
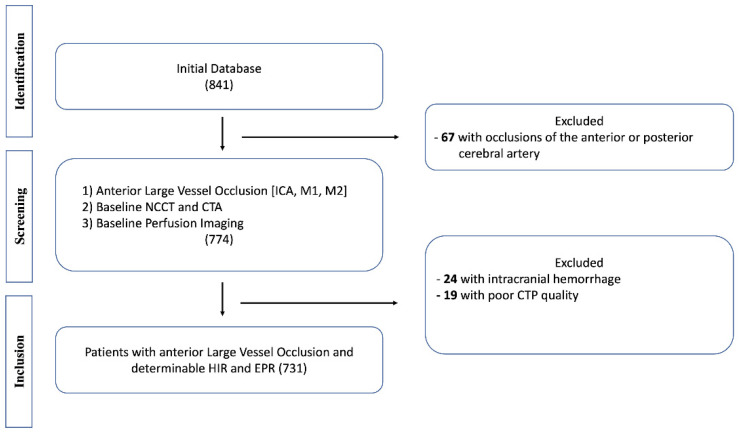
Patient inclusion and exclusion flow chart.

In this study, 731 patients met inclusion criteria (Figure 1). Based on their HIR profiles, 381 patients (52%) were considered to have favorable HIR profiles (HIR+), whereas 350 patients were found to have unfavorable HIR profiles (HIR−).

Fewer patients in the HIR+ group had a history of hypertension compared to the HIR- patients (*n* = 248 (65%) versus *n* = 256 (73%); *p* = 0.024). There were no significant differences with respect to other demographical or medical history data (Table 1).

Compared to patients with unfavorable HIR, HIR+ patients likely had significantly lower median admission NIHSS scores (12 (IQR 7–17) versus 17 (IQR 13–20); *p* < 0.001) and more patients exhibited vessel recanalization either spontaneously or after thrombectomy or intravenous alteplase treatment (*n* = 305 (80%) versus *n* = 248 (71%); *p* < 0.001).

With respect to the admission imaging findings, patients in the HIR+ group had higher median ASPECTS (8 (IQR 7–10) versus 7 (IQR 6–8); *p* < 0.001), lower median baseline infarct core volumes (0 (IQR 0–10) versus 31 (IQR 13–64); *p* < 0.001) and more frequently exhibited favorable arterial CTA collateral profiles (*n* = 304 (79%) versus *n* = 195 [56%]; *p* < 0.001).

**Figure 2:**
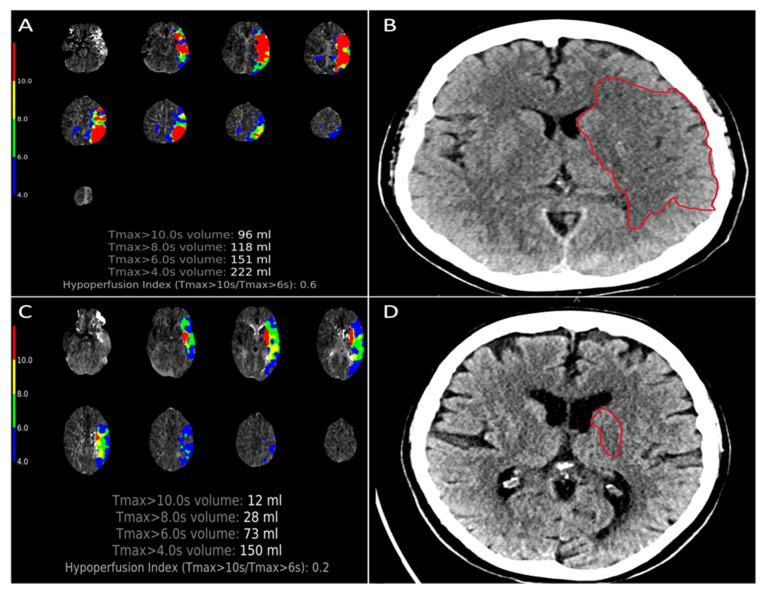
Imaging example of early edema progression in two patients with poor and favorable HIR. Two patients with comparable large vessel occlusion (proximal M1 occlusion) were both treated by endovascular thrombectomy. A 67-year old male patient (**A**,**B**) with symptom onset 2 h before admission imaging showing unfavorable HIR profiles (HIR = 0.6) and a considerable large baseline infarct hypodensity on non-contrast head CT (marked by the red field in (**B**)). The early edema progression rate was calculated at 5.18%/hour for this patient. A 71-year old female patient (**C**,**D**) who was last seen well 3.2 h before admission imaging exhibited only small infarct hypoattenuation in the basal ganglia (marked by the red circle in (**D**)), showing a favorable HIR profiles of 0.2 on admission perfusion imaging. The related early edema progression rate for this patient was calculated at 0.48% per hour.

Patients with a favorable HIR showed lower median ischemic lesion NWU on admission imaging (4% (IQR 2.1–7.6) versus 8.2% (IQR 6–10.4); *p* < 0.001) and reduced EPR (0.016%/h (IQR 0.007–0.04) versus 0.044%/hour (IQR 0.021–0.089); *p* < 0.001) compared to those with unfavorable HIR profiles (Figure 2). Patients in the HIR+ group had lower median scores on the mRS90 compared to HIR- patients (median 2 (IQR 1–5) versus median 4 (IQR 3–6); *p* < 0.001) (Table 2).

**Figure 3:**
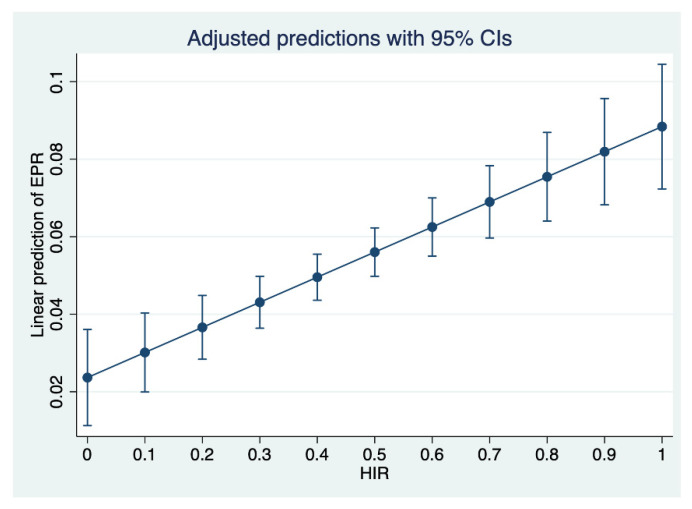
Adjusted prediction of early edema progression by HIR. A multivariable logistic regression plot was used to demonstrate the linear prediction of early edema progression rate (EPR, *y* axis) based on the hypoperfusion intensity ratio (HIR, *x* axis). EPR = Early Edema Progression Rate; HIR = Hypoperfusion Intensity Ratio; 95% CI = 95% Confidence Interval.

For the assessment of the primary outcome (EPR), a multivariable linear regression model was performed. HIR was found to be non-normally distributed. However, as our sample size was large enough, a multivariable linear regression model was deemed suitable for the assessment of the primary study outcome. Furthermore, we found a weak but significant correlation between HIR and NIHSS (r = 0.35; *p* = <0.001), ASPECTS (r = −0.33; *p* < 0.001), CTA collaterals (r = −0.33; *p* < 0.001), and occlusion location (r = −0.198, *p* < 0.001) when accounting for the collinearity of the included variables in the model. These findings were similar to other previous studies on HIR, and as the variance of the inflation factor was found to be low (<2), the variables were included into the multivariable linear model. We found that favorable HIR (*β*: 0.53, SE: 0.02; *p* = 0.003) and presentation NIHSS scores (*β*: 0.2, SE: 0.0006; *p* = 0.001) were independently associated with EPR, regardless of the ASPECTS score on admission NCCT (*β*: −0.11, SE: 0.002; *p* = 0.551), arterial CTA collaterals (*β*: −0.46, SE: 0.008; *p* = 0.579), proximal vessel occlusion localization (*β*: 0.03, SE: 0.0034; *p* = 0.349), blood glucose (*β*: 0.0004, SE: 0; *p* = 0.62), age (*β*: 0.0013, SE: 0.0002; *p* = 0.604), and sex (*β*: −0.0084, SE: 0.007; *p* = 0.229) (Table 3 and Figure 3).

## 4. Discussion

In this study of AIS-LVO patients anticipated for thrombectomy treatment, we aimed to investigate whether imaging biomarkers of cerebral tissue perfusion are associated with early brain edema formation. We found that favorable HIR profiles, defined as a HIR of 0.4, were independently associated with decreased ischemic lesion NWU on admission imaging and a reduced EPR, defined as relative NWU per hour from symptom onset to imaging. Our findings underline the clinical potential of perfusion imaging biomarkers as surrogates for the assessment of infarct growth and brain edema development prior to treatment.

On a larger scale, our observations that favorable HIR profiles were found to be associated with less edema formation and reduced EPR at admission support the assumption that favorable collateral blood flow preserves tissue viability in ischemic brains after the occlusion of a large vessel. This hypothesis is further strengthened by our finding that favorable HIR profiles were correlated to favorable baseline clinical and imaging characteristics, i.e., presentation NIHSS and ASPECTS on admission. HIR is a standardized measure, which is automatically calculated from perfusion imaging [6]. Favorable HIR profiles were reported to correlate with robust arterial collateral status and robust microvascular blood transit through ischemic tissue, both of which are related to edema formation within ischemic brains [7,8,9,16,17,18].

These observations are similar to those of Broocks et al. [16], who also found higher admission ASPECTS, lower admission NIHSS, better median mRS scores after three months, and higher vessel recanalization rates in patients with good arterial collateral scores and less edema formation. Interestingly, HIR, but not pial arterial collateral status, was independently associated with reduced brain edema in our study in the multivariable regression analysis. In contrast, while most HIR+ patients also exhibited robust pial arterial collaterals, approximately 20% of patients were found to have favorable HIR profiles but poor arterial collateralization (Table 2). To date, it is not completely understood why some AIS-LVO patients may exhibit robust cerebral tissue perfusion, despite the lack of pial arterial collaterals and vice versa. One explanation for that may be that both parameters, although somewhat interrelated, assess different levels of microvascular blood flow in ischemic brains. While the arterial collateral status is associated with blood transit to the ischemic tissue, HIR is believed to be more likely related to microvascular blood transit on a tissue level (so called tissue-level collaterals), which differs substantially from the findings of previous studies [12,16]. Another explanation may be that the temporal recruitment of tissue-level collaterals differs from pial collaterals during the time-course of AIS-LVO, e.g., due to local autoregulation of the brain or aggravated progressive brain edema, thus leading to divergent imaging findings [12,19]. This assumption may further be supported by the finding of another study, which found that favorable HIR, but not arterial collaterals, was correlated to delayed edema formation 48–72 h after thrombectomy treatment [7]. Brain edema formation follows a non-linear pattern [16] and the robustness of “collateral-governed” microvascular perfusion in ischemic brains is known to vary over time, which may lead to the assumption that some collateral parameters may influence the tissue fate in earlier stages of ischemia, while other collateral biomarkers more strongly influence the long-term perseverance of ischemic brain tissue [20,21,22,23,24,25]. However, as demonstrated in previous studies, HIR seems to be strongly associated with edema formation, infarct progression, and long-term clinical outcomes after thrombectomy treatment [6,7,11]. Thus, it is conceivable that HIR may have strong prognostic implications regarding the procedural and clinical outcomes of AIS-LVO patients anticipated for thrombectomy treatment. Further studies are needed to better understand the timely mechanisms that alter microvascular collateral perfusion with respect to cerebral brain edema development over time and the specific mechanisms that influence clinical recovery after treatment.

Our study has several limitations. First, the retrospective design of the study may introduce bias. Next, the generalizability of our results may be limited, due to the specific utilized study protocol. To date, NWU determination is still time-consuming and laborious, thus limiting the broader applicability of this approach. There is still no standardized or automated approach for the determination of pial arterial collaterals on CT angiography images, which may introduce bias. Lastly, CT perfusion is still not available at many primary and secondary care centers, which limits the practicability of our approach.

Lastly, different thresholds for the dichotomization of HIR exist in the literature, and further research is needed to identify the optimal HIR threshold for the prediction of early edema development. Multicollinearity remains a potential problem in studies examining the impact of biomarkers that share a common pathophysiology. We tested for multicollinearity and found the intercorrelation between HIR and other co-variables to be low; however, we cannot fully exclude potential bias from multicollinearity.

## 5. Conclusions

Favorable HIR profiles were associated with less ischemic edema formation and reduced temporal edema development from symptom onset to imaging in AIS-LVO patients.

## Figures and Tables

**Table 1:** Patient characteristics and stroke presentation details dichotomized by the hypoperfusion intensity ratio ^a^.

	HIR+ (*n* = 381)	HIR− (*n* = 350)	*p*-Value
Age (years), median (IQR)	75 (64–82)	76 (64–84)	0.562
Female	200 (52.2)	176 (50.3)	0.551
**Medical History**			
Arterial Fibrillation	146 (38.1)	141 (40.3)	0.645
Hypertension	248 (64.8)	256 (73.1)	0.024
Blood Glucose, median (IQR)	120 (105–146)	123 (104–148)	0.657
Diabetes Mellitus	66 (18.9)	85 (22.2)	0.235
**Smoking**			
Current Smoker	42 (11)	41 (11.7)	0.997
Never Smoked	245 (64)	246 (70.3)	0.571
Prior Smoker	61 (15.9)	53 15.1)	0.494
Unknown Smoking Status	33 (8.7)	10 (2.9)	
**Stroke Presentation Details**			
Presentation NIHSS, median (IQR)	12 (7–17)	17 (13–20)	<0.001
Time from Symptom Onset to i.v. tPA in min, median (IQR)	95 (73–164)	108 (66–150)	0.838
Time from Symptom Onset to imaging, min, median (IQR)	197 (100–396)	160 (86–335)	0.61
**Treatment Details**			
**Intravenous tPA Details**			
i.v. tPA Administration	198 (51.7)	168 (48)	0.343
**Endovascular Treatment Details**			
**Treated by endovascular thrombectomy**	342 (89.8)	348 (99.4)	0.58
Recanalization after thrombectomy (TICI 2b-3)	287 (83.9)	270 (77.5)	0.01
**Vessel Recanalization by tPA, Thrombectomy or Spontaneous**	305 (79.6)	248 (70.9)	0.001

Patient characteristics and stroke presentation details of all 731 patients dichotomized by favorable (HIR+) and unfavorable (HIR−) hypoperfusion intensity ratios. Values are displayed as absolute numbers and frequencies, mean ± SD or median and interquartile range (IQR). The univariate logistic regression analysis between HIR+ and HIR− for all parameters is displayed on the very right column with corresponding *p*-values. NIHSS = National Institutes of Health Stroke Scale; i.v. tPA = intravenous tissue plasminogen activator; TICI = thrombolysis in cerebral infarction. ^a^ Data are *n* (%), unless otherwise indicated.

**Table 2:** Presentation imaging details and clinical outcome dichotomized by the hypoperfusion intensity ratio ^a^.

	HIR+ (*n* = 381)	HIR− (*n* = 350)	*p* Value
ASPECTS, median (IQR)	8 (7–10)	7 (6–8)	<0.001
Baseline Infarct Core Volume [CBF < 30%] (mL), median (IQR)	0 (0–10)	31 (13–64)	<0.001
Penumbra Tmax > 6 s volume (mL), median (IQR)	90.5 (54.5–140)	145 (88–197)	<0.001
Penumbra Tmax > 10 s volume (mL), median (IQR)	20.8 (8–45.2)	89.6 (49.7–126)	<0.001
NWU on admission (%), median (IQR)	4 (2.1–7.6)	8.2 (6–10.4)	<0.001
Early Edema Progression Rate, (%/h), median (IQR)	0.96 (0.42–2.4)	2.64 (1.26–5.34)	<0.001
Hypoperfusion intensity ratio (HIR), median (IQR)	0.2 (0.1–0.3)	0.6 (0.5–0.7)	<0.001
Favorable CTA collaterals (TAN)	304 (79.4)	195 (55.7)	<0.001
Vessel occlusion localization on CTA			
Internal carotid artery	60 (15.7)	87 (24.9)	0.004
Proximal MCA 1 segment occlusion	119 (31.1)	153 (43.7)	<0.001
Distal MCA 1 segment occlusion	103 (26.9)	59 (16.9)	0.001
MCA 2 segment occlusion	96 (25.1)	51 (14.6)	0.001
Long-term clinical outcome			
Modified Ranking Scale after 90 days, median (IQR)	2 (1–5)	4 (3–6)	<0.001
Unknown	19 (5)	8 (2.3)	

Details of baseline imaging and functional outcome measures of all 731 patients dichotomized by favorable (HIR+) and unfavorable (HIR−) hypoperfusion intensity ratios. Values are displayed as absolute numbers and frequencies, mean ± SD or median and interquartile range (IQR). Univariate logistic regression analysis between HIR+ and HIR− for all parameters is displayed on the very right column with corresponding *p*-values. ASPECTS = Alberta Stroke Program Early CT Score; CBF = cerebral blood flow; Tmax > 6 = time-to-maximum of the tissue residue function with a delay of >6 s; Tmax > 10 = time-to-maximum of the tissue residue function with a delay of >10 s; NWU = Net Water Uptake; MCA = Middle Cerebral Artery. Favorable CTA collaterals were defined as TAN > 50%, assessed on CT angiography. ^a^ Data are *n* (%), unless otherwise indicated.

**Table 3:** Linear multivariable regression analysis for primary outcome (EPR).

	Early Edema Progression Rate		
*Predictors*	*β*	*SE*	*p-Value*
Favorable HIR	0.53	0.017	0.003
Presentation NIHSS	0.2	0.0006	0.001
Baseline ASPECTS	−0.11	0.002	0.551
Favorable TAN collaterals	−0.46	0.0083	0.579
Proximal vessel occlusion	0.03	0.0034	0.349
Blood glucose	0.00036	0	0.62
Age	0.0013	0.0002	0.604
Sex	−0.0084	0.007	0.229
Observations *n* = 701			

Calculated Beta (β), Standard error (SE) and *p*-value of all available (*n* = 701) patients screened via multivariable linear regression analysis. *n* = 30 were excluded from this analysis due to unknown blood glucose values. EPR = early edema progression rate; HIR = hypoperfusion intensity ratio; NIHSS = National Institute and Health Stroke Scale; ASPECTS = Alberta Stroke Program Early CT Score; NCCT = non-contrast head computed tomography. Proximal vessel occlusion is regarded as presence of anterior circulation large vessel occlusion of the internal carotid artery or first (M1) or second (M2) segment of the middle cerebral artery. Favorable HIR is defined as HIR < 0.4; favorable CTA collaterals were defined as TAN > 50%, assessed on CT angiography.

## Data Availability

The data that support the findings of this study are available from the corresponding author upon reasonable request.
